# Supplementary material for: Red Meat Derived Glycan, N-acetylneuraminic Acid (Neu5Ac) Is a Major Sialic Acid in Different Skeletal Muscles and Organs of Nine Animal Species—A Guideline for Human Consumers
Source: Foods. 2023 Jan 10;12(2):337. doi: 10.3390/foods12020337 (PMC9858279; doi:10.3390/foods12020337)
Supplement: Supplementary file 1 [file foods-12-00337-s001.zip › foods-2110290-supplementary.pdf]

Table S1 Information on animals of different species regarding breed, age and gender.

Species	Animal no	Breed	Age	Gender
Sheep (*Ovis aries*)	1	Merino	5 y	Female
	2	Dorper	2.5 y	Female
	3	Merino	2 y	Female
	4	Merino	5 y	Female
	5	Merino	10 mo	Female
	6	Merino	> 5 y	Female
	7	Dorper	> 5 y	Female
	8	Merino	> 5 y	Female
	9	Merino	> 5 y	Female
	10	Merino	> 5 y	Male
Cattle (*Bos Taurus*)	1	Red Angus	1 y	Female
	2	Hereford	6-7 y	Female
	3	Hereford	5 y	Female
Goat (*Capra aegagrus hircus)*	1	Feral	1 y	Female
	2	Boer Goat	2 y	Female
	3	Boer Goat	6 mo	Male
Pig (*Sus scrofa*)	1	Landrace × Large White cross	6 mo	Male
	2	Landrace × Large White cross	8 mo	Male

	3	Landrace × Large White cross	8 mo	Male
Deer (*Odocoileus virginianus)*	1	Rusa	1 y	Male
	2	Rusa	2-3 y	Female (pregnant)
	3	Rusa	1 y	Female
Horse (*Equus caballus*)	1	Appaloosa	18 y	Female
	2	Welsh Pony	3 y	Female
	3	Appaloosa	21 y	Female
Kangaroo (*Macropus giganteus*)	1	Eastern grey	unknown	Female
	2	Eastern grey	unknown	Female
	3	Eastern grey	unknown	Female
Cat (*Felis catus*)	1	Domestic short hair	3 y	Male
	2	Domestic short hair	3 y	Female
	3	Domestic short hair	6 mo	Female
Dog (*Canis lupus familiaris*)	1	Great Dane X	2 y	Male
	2	Pit bull X	4 y	Male
	3	Red heeler X	4 y	Male

A

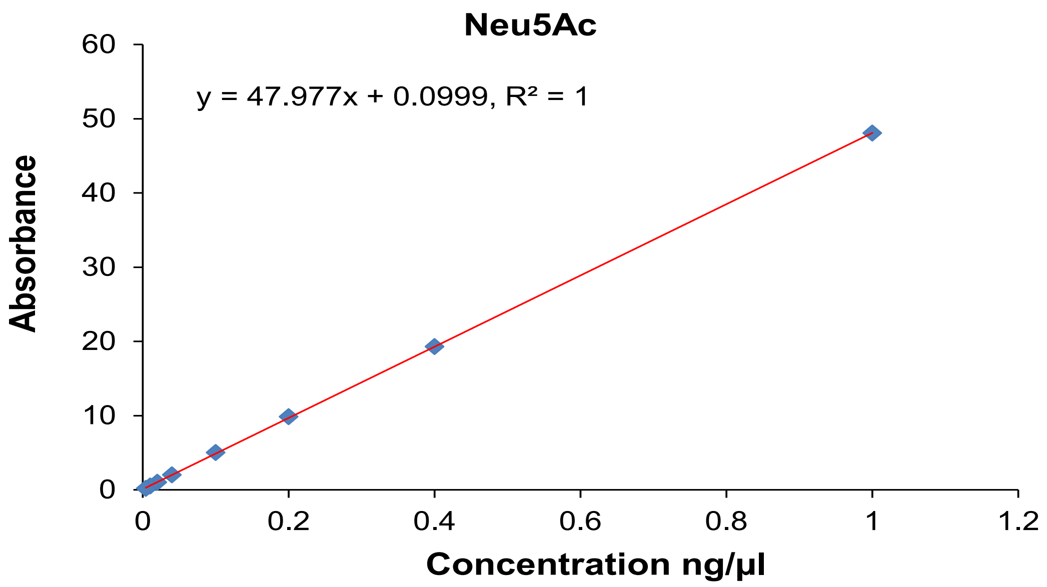

B

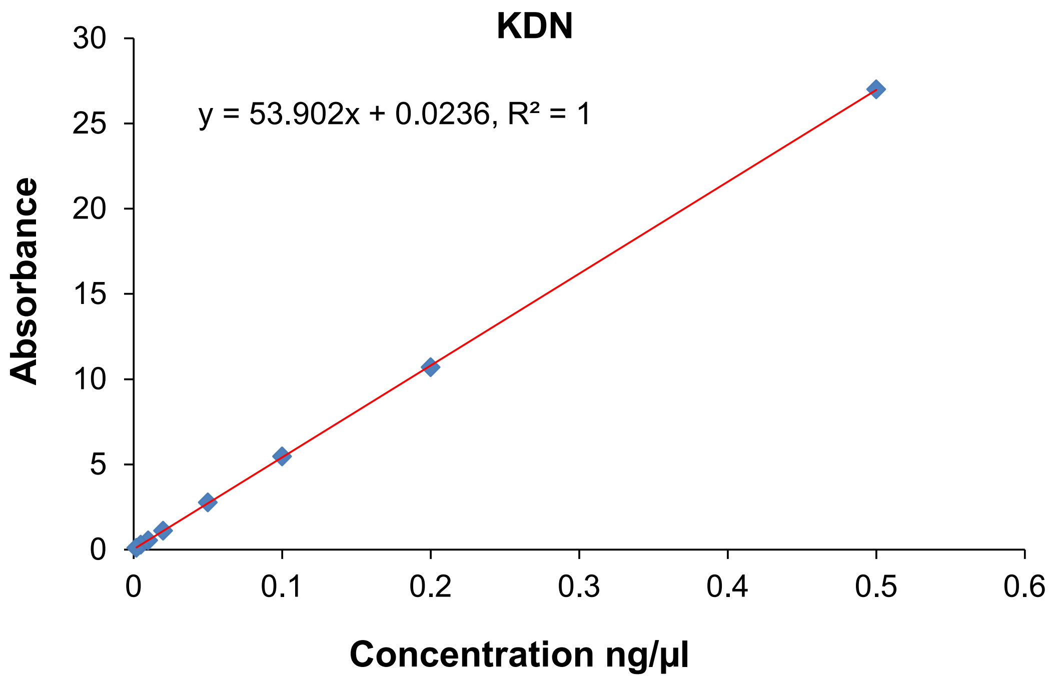

Figure S1. Standard curve for A. Neu5Ac in the concentration range of 0.004-1 ng/µl nm B. KDN in the concentration range of 0.002- 0.5 ng/µl at 448 emissions and 373nm excitation.

A

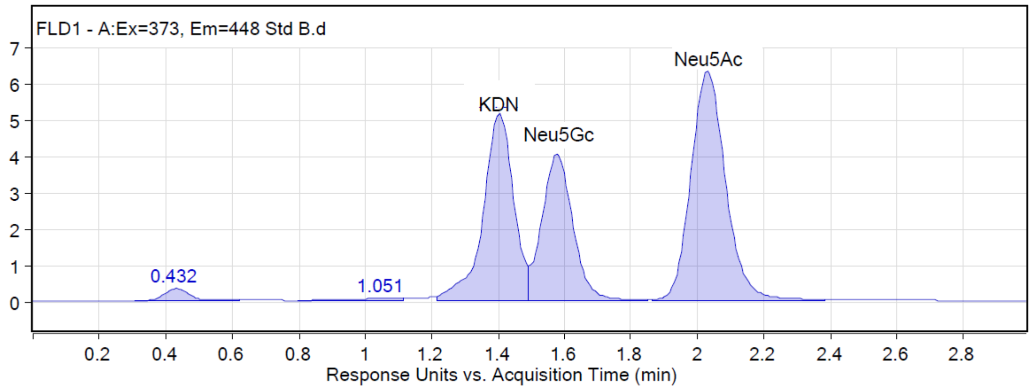

Figure S2. UHPLC trace for mixed standard of *N*-acetylneuraminic acid (Neu5Ac), and 2-keto-3-deoxy-D-glycero-D-galacto-nononic acid (KDN).

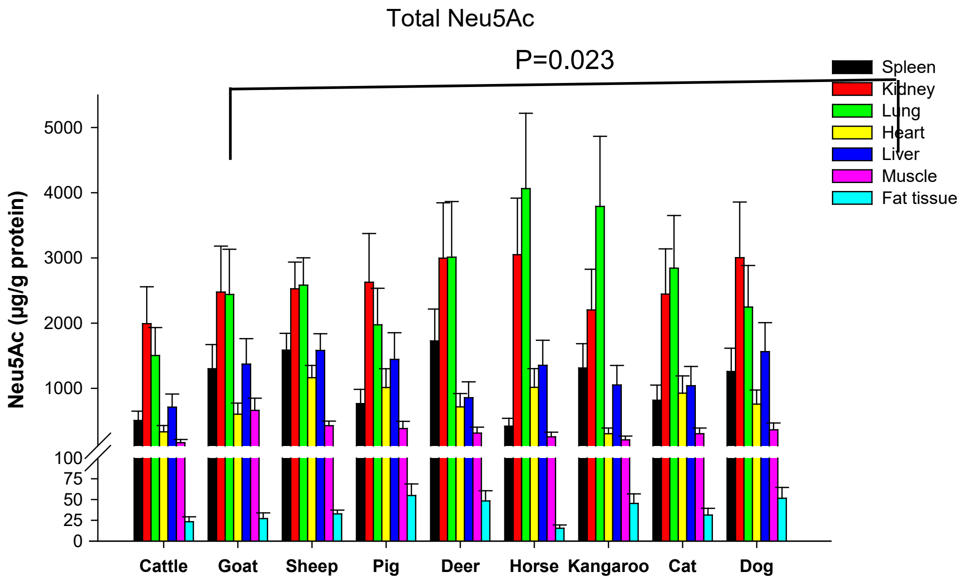

Figure S3. Comparison of total Neu5Ac concentration in the 5 organ tissues and skeletal muscle of 9 animal species (n=10 for sheep and n=3 for other animal species, linear mixed models). Significant differences were observed among difference species for all samples comparisons (P=0.023 species X sample interaction). Values expressed as μg/g lipid for fat tissue only. Values are mean ± SEM.

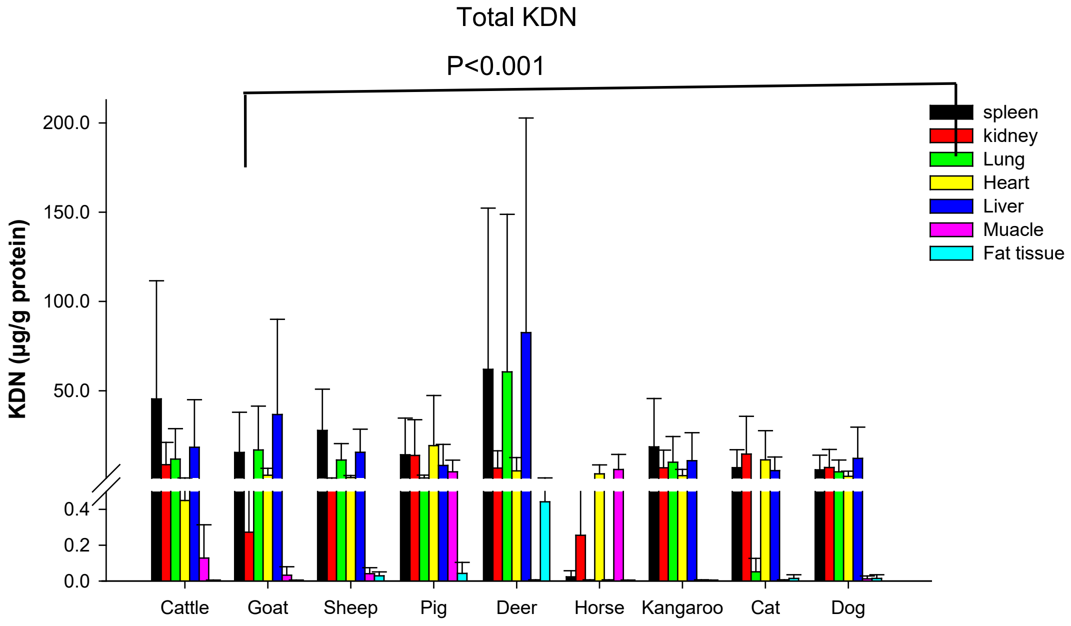

Figure S4. Comparison of total KDN concentration in the organ tissues and skeletal muscle of 9 different animal species (n=10 for sheep and n=3 for other animal species, linear mixed model). Significant differences were observed among difference species for all samples comparisons (P<0.001), based on species x sample interaction. Values expressed as µg/g lipid for fat tissue only. Values are mean ± SEM.
